# Supplementary material for: Rationally Designed Novel Antimicrobial Peptides Targeting Chitin Synthase for Combating Soybean Phytophthora Blight
Source: Int J Mol Sci. 2024 Mar 20;25(6):3512. doi: 10.3390/ijms25063512 (PMC10971155; doi:10.3390/ijms25063512)
Supplement: Supplementary file 1 [file ijms-25-03512-s001.zip › ijms-2915575-supplementary.pdf]

Supporting Information

Rationally Designed Novel Antimicrobial Peptides Targeting Chitin Synthase for Combating Soybean Phytophthora Blight

Yue Ran, Kiran Shehzadi, Jian-Hua Liang * and Ming-Jia Yu *

School of Chemistry and Chemical Engineering, Beijing Institute of Technology, Beijing 100081, China
* Correspondence: ljhbit@bit.edu.cn (J.-H.L.); mjyu@bit.edu.cn (M.-J.Y.)

Contents

Table S1: Library of antimicrobial peptides with inhibitory activity against *Phytophthora*.. 2

Table S2: The interaction of hydrogen bonds between AMP_04 and chitin synthase.. 3

Table S3: The interaction of hydrogen bonds between DP and chitin synthase. 3

Table S4: The interaction of hydrogen bonds between TP and chitin synthase.. 4

Figure S1: The interaction between the receptor (in deep blue) and the ligand displayed by LigPlot+.. 5

Figure S2: Molecular dynamics simulation results between TP and chitin synthase.. 6

Figure S3: Predicting plDDT plots of four antimicrobial peptides using alphafold2.. 7

Table S1. Library of antimicrobial peptides with inhibitory activity against *Phytophthora.*

Serial number	Amino acid sequence	Plant pathogen	Inhibition efficacy	Reference
01	NH_2-GIGKFLHSAKKFGKAF VGEIMNS-COOH	*Phytophthora infestans*	MIC=40 (μg/ml)	1
02	NH_2-GLWGTLCGLFGWLGA AGKTTGSARAKQRLKLMAG -COOH	*Phytophthora infestans*	MIC=20 (μg/ml)	1
03	NH_2-KWKVFKKIEKMGRNIR NGIVKAGPAIAVLGEAKAL-COOH	*Phytophthora infestans*	MIC=20 (μg/ml)	1
04	NH_2-EWKLLKKIGAVLKLV-COOH	*Phytophthora infestans*	MIC=15 (μM)	2
05	NH_2-KWKLFKKILKVL-COOH	*Phytophthora infestans*	MIC=15 (μM)	2
06	NH_2-WKLFKKILKVL-COOH	*Phytophthora infestans*	MIC=15 (μM)	2
07	NH_2-KWKLFKKIGIGAVLKV LTTGLPALIS-COOH	*Phytophthora infestans*	MIC=40 (μM)	2
08	NH_2-GIFSKLAGKKLKNLLIS GL-COOH	*Phytophthora nicotianae*	MIC=12 (μM)	3
09	NH_2-GIFSKLAGKKLKNLLIS GLKNVGKEVGLDVVRTGIDI AGCKIKGEC-COOH	*Phytophthora nicotianae*	MIC=75 (μM)	3
10	NH_2-EGPVGLADPDGPASAP LGAP-COOH	*Phytophthora capsici*	EC_{50}=3.14 (μg/ml)	4
11	NH_2-FRLKFH-COOH	*Phytophthora infestans*	IC_{50}=56 (μM)	5
12	NH_2-RLARLAR-COOH	*Phytophthora infestans*	IC_{50}=910 (μM)	5
13	NH_2-WKLFKKILKVL-COOH	*Phytophthora infestans*	IC_{50}=17 (μM)	5
14	NH_2-MASRAARLAARLARLA LRAL-COOH	*Phytophthora infestans*	IC_{50}=14 (μM)	5
15	NH_2-YSYKKIDCGGACAARO RLSSRPRLCNRACGTCCARC NCVPPGTSGNTETCPCYASL TTHGNKRKCP-COOH	*Phytophthora cucumerina*	EC_{50}=10 (μM)	6
16	NH_2-GSNFCDSKCKLRCSKA	*Phytophthora cucumerina*	EC_{50}=10 (μM)	6

GLADRCLKYCGICOEECKVV
PSGTYGNKHECPCYRDKKN
SKGKSKCP-COOH

Table S2. The interaction of hydrogen bonds between AMP_04 and chitin synthase.

Antimicrobial peptide	Receptor residue	Ligand residue	Hydrogen bond [Å]	Hydrogen bond angle [°]
AMP_04	r:SER534	l:ASP8	2.4064	131.532
	r:ASP382	l:ALA13	2.2816	149.906
	r:PRO454	l:SER14	1.9174	173.496
	r:ASP440	l:GLY18	2.5774	119.957
	r:LYS355	l:GLY2	1.9231	143.658
	r:SER436	l:PRO16	2.1238	157.535
	r:SER436	l:LEU17	2.3747	100.13
	r:SER451	l:PRO20	2.5826	160.587
	r:ARG497	l:PRO20	2.0833	129.641
	r:LYS537	l:LEU6	2.4322	135.489
	r:ARG538	l:GLU1	2.7702	118.131
	r:ARG538	l:PRO9	2.5881	130.017
	r:ARG538	l:PRO3	2.299	116.71
	r:TRP539	l:ALA13	2.9607	106.998
	r:TYR492	l:PRO3	2.4986	140.745
	r:ASP524	l:PRO12	3.0166	128.342
	r:ASP382	l:PRO12	2.623	143.675
	r:PRO454	l:ALA13	2.6924	130.504
	r:ARG538	l:ASP8	3.0407	118.905

Table S3. The interaction of hydrogen bonds between DP and chitin synthase.

Antimicrobial peptide	Receptor residue	Ligand residue	Hydrogen bond [Å]	Hydrogen bond angle [°]
AMP_04_D10k_S14L (DP)	r:SER534	l:ASP8	2.4061	131.554
	r:VAL383	l:LYS10	2.7573	125.104
	r:GLU241	l:LYS10	2.2056	153.118
	r:GLN245	l:LYS10	2.0885	120.851
	r:ASP382	l:ALA13	2.2695	149.456
	r:PRO454	l:LEU14	1.9228	170.018
	r:ASP440	l:GLY18	2.5774	119.957
	r:LYS355	l:GLY2	1.9236	143.631
	r:SER436	l:PRO16	2.1215	157.315
	r:SER436	l:LEU17	2.3509	101.574
	r:SER451	l:PRO20	2.5726	160.775

	r:ARG497	l:PRO20	2.0696	129.543
	r:ARG497	l:ALA19	2.8065	164.996
	r:LYS537	l:LEU6	2.4322	135.489
	r:ARG538	l:GLU1	2.8071	116.936
	r:ARG538	l:PRO9	2.5597	131.470
	r:ARG538	l:PRO3	2.3190	117.634

Table S4. The interaction of hydrogen bonds between TP and chitin synthase.

**Antimicrobial peptide**	**Receptor residue**	**Ligand residue**	**Hydrogen bond [Å]**	**Hydrogen bond angle [°]**
AMP_04_D10K_G11I_S14L (TP)	r:SER534	l:ASP8	2.4062	131.540
	r:VAL383	l:LYS10	2.3032	140.361
	r:GLU241	l:LYS10	2.0644	149.860
	r:GLN245	l:LYS10	2.2089	124.333
	r:ASP382	l:ALA13	2.2670	148.960
	r:PRO454	l:LEU14	1.9229	169.958
	r:ASP440	l:GLY18	2.5774	119.957
	r:LYS355	l:GLY2	1.9236	143.620
	r:SER436	l:PRO16	2.1211	157.251
	r:SER436	l:LEU17	2.3506	101.568
	r:SER451	l:PRO20	2.5739	160.843
	r:ARG497	l:PRO20	2.0620	130.102
	r:ARG497	l:A19	2.8067	165.384
	r:LYS537	l:LEU6	2.4301	135.709
	r:ARG538	l:GLU1	2.9462	114.847
	r:ARG538	l:PRO9	2.4624	134.716
	r:ARG538	l:PRO3	2.4198	118.125
	r:ARG538	l:GLU1	3.0346	114.473
	r:TRP539	l:ALA13	2.8935	116.991

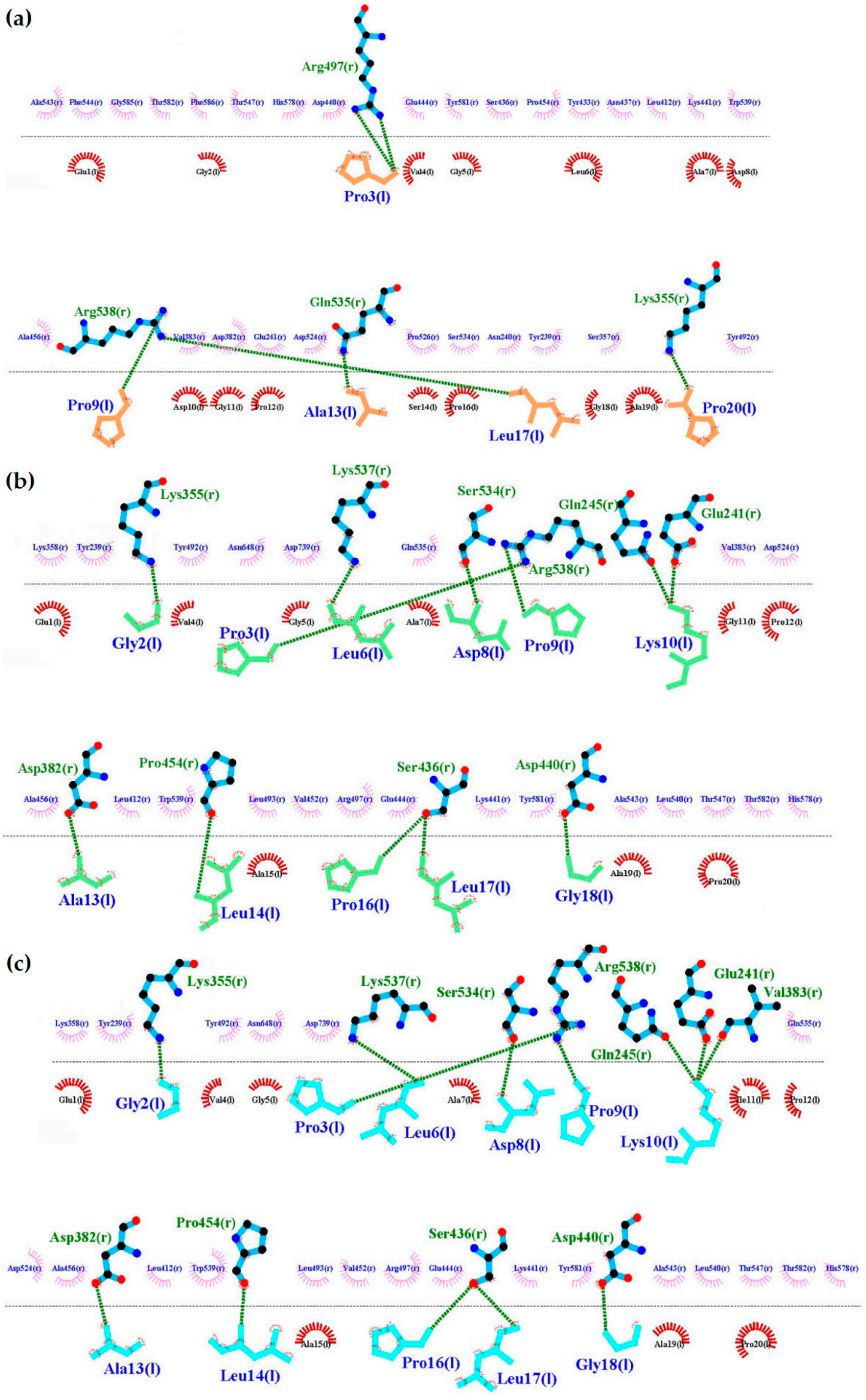

Figure S1. The interaction between the receptor (in deep blue) and the ligand displayed by LigPlot+. (a) The 2D interaction map between the receptor and AMP_04, with AMP_04 depicted in orange. (b) The 2D interaction map between the receptor and the double mutant DP, with DP represented in green. (c) The 2D interaction map between the receptor and the triple mutant TP, with TP shown in sky blue.

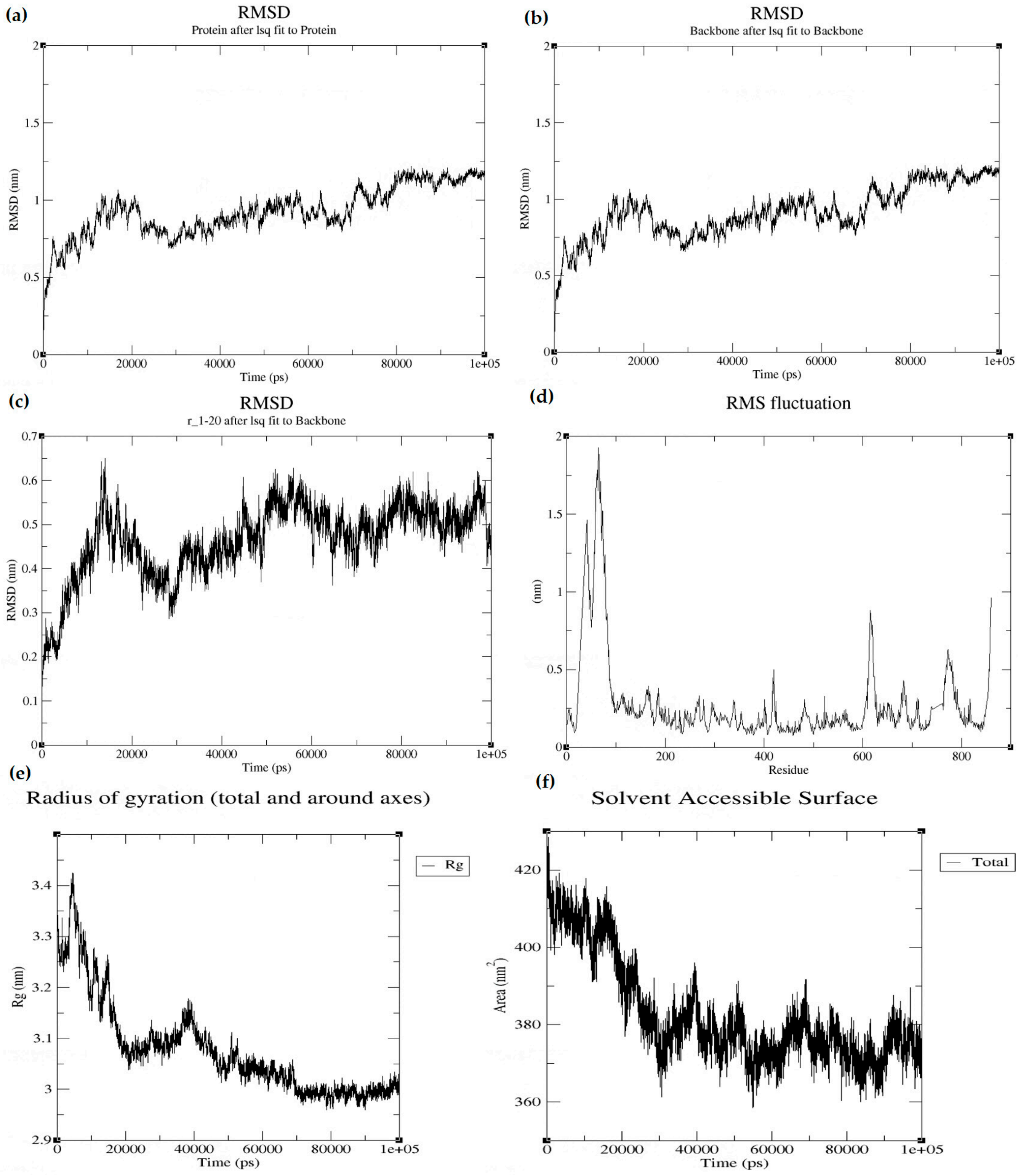

Figure S2 Molecular dynamics simulation results between TP and chitin synthase. (a) Overall RMSD plot of the receptor-lig and protein. (b) RMSD plot of the receptor-ligand backbone. (c) RMSD plot of the antimicrobial peptide TP. (d) RMSF plot of the overall receptor-ligand protein. (e) Gyration radius plot of the overall receptor-ligand protein. (f) Solvent accessible s urface area plot of the overall receptor-ligand protein.

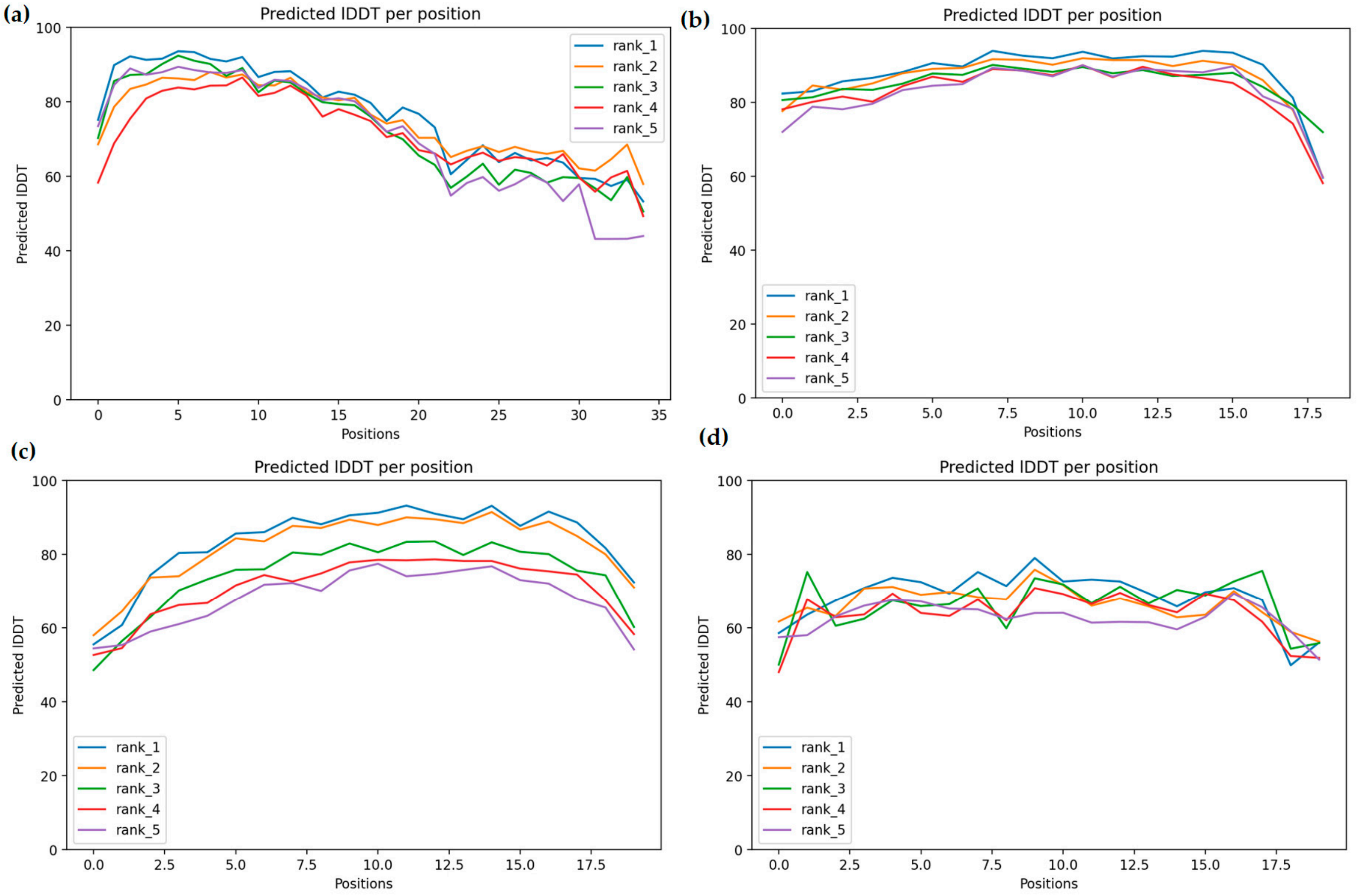

Figure S3 Predicting plDDT plots of four antimicrobial peptides using alphafold2. (a) AMP_01. (b) AMP_02. (c) AMP_03. (d) AMP_04.

Reference

[1] A. R. Alan and E. D. Earle, Sensitivity of bacterial and fungal plant pathogens to the lytic peptides, MSI-99, magainin II, and cecropin B, *Mol Plant Microbe Interact*, 2002, **15**, 701-708.

[2] L. Cavallarin, D. Andreu and B. San Segundo, Cecropin A-derived peptides are potent inhibitors of fungal plant pathogens, *Mol Plant Microbe Interact*, 1998, **11**, 218-227.

[3] M. L. Mangoni, D. Fiocco, G. Mignogna, D. Barra and M. Simmaco, Functional characterisation of the 1-18 fragment of esculentin-1b, an antimicrobial peptide from Rana esculenta, *Peptides*, 2003, **24**, 1771-1777.

[4] Y. Xiao, F. Meng, D. Qiu and X. Yang, Two novel antimicrobial peptides purified from the symbiotic bacteria Xenorhabdus budapestensis NMC-10, *Peptides*, 2012, **35**, 253-260.

[5] G. S. Ali and A. S. Reddy, Inhibition of fungal and bacterial plant pathogens by synthetic peptides: in vitro growth inhibition, interaction between peptides and inhibition of disease progression, *Mol Plant Microbe Interact*, 2000, **13**, 847-859.

[6] V. Bartova, J. Barta and M. Jarosova, Antifungal and antimicrobial proteins and peptides of potato (Solanum tuberosum L.) tubers and their applications, *Appl Microbiol Biotechnol*, 2019, **103**, 5533-5547.
